# SARS-CoV-2 Rapid Antigen Test Based on a New Anti-Nucleocapsid Protein Monoclonal Antibody: Development and Real-Time Validation

**DOI:** 10.3390/microorganisms11102422

**Published:** 2023-09-28

**Authors:** Fabiana Fioravante Coelho, Miriam Aparecida da Silva, Thiciany Blener Lopes, Juliana Moutinho Polatto, Natália Salazar de Castro, Luis Adan Flores Andrade, Karine Lima Lourenço, Hugo Itaru Sato, Alex Fiorini de Carvalho, Helena Perez Coelho, Flávia Fonseca Bagno, Daniela Luz, Vincent Louis Viala, Pedro Queiroz Cattony, Bruna de Sousa Melo, Ana Maria Moro, Wagner Quintilio, Ana Paula Barbosa, Camila Gasque Bomfim, Camila Pereira Soares, Cristiane Rodrigues Guzzo, Flavio Guimarães Fonseca, Edison Luiz Durigon, Ricardo Tostes Gazzinelli, Santuza M. Ribeiro Teixeira, Roxane Maria Fontes Piazza, Ana Paula Fernandes

**Affiliations:** 1 Faculdade de Farmácia, Universidade Federal de Minas Gerais, Belo Horizonte 31270-901, Brazil; ffioravante23@gmail.com; 2 Hospital da Polícia Militar de Minas Gerais, Polícia Militar de Minas Gerais, Belo Horizonte 30110-013, Brazil; 3 Centro de Tecnologia em Vacinas, Universidade Federal de Minas Gerais, Belo Horizonte 31310-260, Brazil; thicy_lopes@hotmail.com (T.B.L.); natsalazar@gmail.com (N.S.d.C.); luisadanflores@gmail.com (L.A.F.A.); karine_lourenco@hotmail.com (K.L.L.); hugo_itaru@hotmail.com (H.I.S.); alexficar@gmail.com (A.F.d.C.); helenaperezcoelho@gmail.com (H.P.C.); flavia.bagno@gmail.com (F.F.B.); fdafonseca@icb.ufmg.br (F.G.F.); ricardo.gazzinelli@fiocruz.br (R.T.G.); santuzat@ufmg.br (S.M.R.T.); 4 Instituto Butantan, São Paulo 05503-900, Brazil; miriam.silva@butantan.gov.br (M.A.d.S.); juliana.yassuda@butantan.gov.br (J.M.P.); daniela.luz@butantan.gov.br (D.L.); vincent.viala@butantan.gov.br (V.L.V.); pedro.cattony@butantan.gov.br (P.Q.C.); brn.smelo@gmail.com (B.d.S.M.); ana.moro@butantan.gov.br (A.M.M.); wagner.quintilio@butantan.gov.br (W.Q.); ana2.barbosa@usp.br (A.P.B.); roxane.piazza@butantan.gov.br (R.M.F.P.); 5 Department of Microbiology, Institute of Biomedical Sciences, University of São Paulo, São Paulo 05508-900, Brazil; camila.bomfim@usp.br (C.G.B.); camilasoares350@gmail.com (C.P.S.); crisguzzo@usp.br (C.R.G.); elduri-go@usp.br (E.L.D.)

**Keywords:** SARS-CoV-2, diagnosis, nucleocapsid (N) antigen, IgG2b monoclonal antibody, Ag-RDT development, validation, follow-up study

## Abstract

SARS-CoV-2 diagnostic tests have become an important tool for pandemic control. Among the alternatives for COVID-19 diagnosis, antigen rapid diagnostic tests (Ag-RDT) are very convenient and widely used. However, as SARS-CoV-2 variants may continuously emerge, the replacement of tests and reagents may be required to maintain the sensitivity of Ag-RDTs. Here, we describe the development and validation of an Ag-RDT during an outbreak of the Omicron variant, including the characterization of a new monoclonal antibody (anti-DTC-N 1B3 mAb) that recognizes the Nucleocapsid protein (N). The anti-DTC-N 1B3 mAb recognized the sequence TFPPTEPKKDKKK located at the C-terminus of the N protein of main SARS-CoV-2 variants of concern. Accordingly, the Ag-RDT prototypes using the anti-DTC-N 1B3 mAB detected all the SARS-CoV-2 variants—Wuhan, Alpha, Gamma, Delta, P2 and Omicron. The performance of the best prototype (sensitivity of 95.2% for samples with Ct ≤ 25; specificity of 98.3% and overall accuracy of 85.0%) met the WHO recommendations. Moreover, results from a patients’ follow-up study indicated that, if performed within the first three days after onset of symptoms, the Ag-RDT displayed 100% sensitivity. Thus, the new mAb and the Ag-RDT developed herein may constitute alternative tools for COVID-19 point-of-care diagnosis and epidemiological surveillance.

## 1. Introduction

The CoronaVirus Disease 2019 (COVID-19), caused by the new coronavirus named Severe Acute Respiratory Syndrome CoronaVirus 2 (SARS-CoV-2), has caused more than 770 million infections and more than 6.9 million deaths worldwide since it was declared a pandemic [1]. COVID-19 vaccination campaigns were essential for pandemic control, but vaccination has progressed slowly worldwide, and it is estimated that only 5 billion people are fully vaccinated. Although vaccines greatly reduced the incidence of severe COVID-19 and death cases, they do not fully prevent transmission and infection by SARS-CoV-2 [2].

A combination of high infection rates, low vaccine coverage, especially in developing countries, and high virus mutation rates contributed to the appearance of SARS-CoV-2 variants, classified either as variants of concern (VOC) or variants of interest (VOI) [3,4]. VOCs and VOIs accumulated mutations, mainly in the Spike protein, leading to an escape from neutralizing antibodies induced by previous infections or vaccination. The SARS-CoV-2 VOCs Delta and Omicron spread worldwide at a speed never seen before for any other variant. The Gamma variant was detected in Brazil in April 2021 [3] and was replaced by the Delta variant, which circulated in Brazil until December 2021 [4]. The Delta variant accumulated a total of nine mutations at the Spike protein [5]. On 26 November 2021, the Omicron BA.1 variant, which displayed 62 nonsynonymous mutations in its genome, 36 of them in the Spike gene, was described in South Africa. In December 2021, the Omicron BA.1 variant was detected in Brazil [6], quickly replacing the Delta variant.

In the current context, while efficient COVID-19 treatments are not widely available, the world population is not fully vaccinated and pre-existing antibodies induced either by previous infections or vaccination may not be sufficient to protect against the continuous emergence of new VOCs, one of the mainstay measures for COVID-19 control remains the prompt identification of SARS-CoV-2 infection in symptomatic individuals and contact cases. As fast as infected individuals are identified, countermeasures including isolation and case notification should be adopted to reduce transmission. In this scenario, antigen rapid diagnostic tests (Ag-RDTs) have become a highly cost-effective alternative compared to the real-time quantitative polymerase chain reaction (RTq-PCR), the gold standard for COVID-19 diagnosis. If correctly performed and interpreted, Ag-RDT can play a significant role in the continuous efforts for disease control, including its use in primary health attention rooms and self-testing [7,8,9]. It has also proved to be a valuable tool for epidemiologic surveys in field conditions. Consequently, during the pandemic, its use spread worldwide and was incorporated into routine tests for the diagnosis of respiratory virus infections. However, the continuous improvements in Ag-RDT, the target study population, and the continuous emergence of new SARS-CoV-2 variants may influence the performance of Ag-RDT [10].

In contrast to the Spike protein, which is the hotspot for mutations on the SARS-CoV-2 genome due to intense evolutionary pressure to escape host antibody responses, the Nucleocapsid protein (N) is not only more conserved among variants, but is also more abundantly expressed during infection [11]. Therefore, the N protein has become one of the main targets for Ag-RDTs development, and some of them are commercially available. Despite the appearance of SARS-CoV-2 variants, the sensitivity of N-based Ag-RDT remains high, so far [12].

Herein, we describe the engineering process to develop an Ag-RDT based on N protein detection, including a new anti-N monoclonal antibody production and characterization, as well as its validation during an Omicron BA.1 variant outbreak in Brazil under field conditions.

## 2. Materials and Methods

### 2.1. Ethical Approval

This study was conducted in agreement with the Ethical Principles in Human Research, approved by the Research Ethics Committee/UFMG (CAAE: 1686320.0.0000.5149). For animal use, this study was carried out following the recommendations of Ethical Principles in Animal Research, adopted by the Brazilian Council of Animal Experimentation. The Ethical Committee for Animal Research from Butantan Institute approved the research protocol (2715140420).

### 2.2. Populational Study

The nasopharyngeal samples were collected from patients attending the public health system in the city of Guaranésia, a small Brazilian town with 19 k habitants [13], in the state of Minas Gerais, at the border with São Paulo State. Sample collection and testing were performed for citizens with suggestive COVID-19 symptoms. The samples were obtained from 1 December 2022 to 8 February 2022, during the outbreak of the Omicron BA.1 variant in the southeast region of Brazil. All samples (*n* = 939) were submitted to RT-qPCR for the diagnosis of SARS-CoV-2 infection.

According to the epidemiological report released by the public health authorities of Minas Gerais State Health Department (SES/MG), by 8 February 2022, 14,350 citizens of Guaranésia had already been immunized with two doses of the vaccines against COVID-19 [14]. This number corresponds to approximately 75.5% of the total city population. In addition, 16,504 inhabitants had already received at least the first dose of the vaccine (approximately 86.8% of the total population).

### 2.3. Follow-Up Study

Patients (*n* = 38) from Belo Horizonte-Minas Gerais (Brazil) with a diagnosis of COVID-19 by RT-qPCR were monitored for 17 days, since the day of symptoms onset (day 0), from 23 December 2021 to 15 February 2022, to assess the sensitivity of the Ag-RDT in parallel with the RT-qPCR test, according to the reported number of days of symptoms of the patient. At least two samples from the 38 patients were collected throughout the infection. The follow-up study totaled 112 positive samples collected. Of the 38 individuals included in this cohort, 84.4% received two vaccine doses against COVID-19, 9.4% received three doses and 3.0% received only one dose. The administration of RNA vaccines was predominant (52%) among these patients, followed by viral vector vaccines (29%) and inactivated vaccines (19%). Samples were assayed with prototype 2.

#### 2.3.1. Sampling

For each patient, two nasopharyngeal swabs were collected, one for Ag-RDT and the other for RT-qPCR, from each nostril. Samples for Ag-RDT were collected with nylon swabs in 0.3 mL of inactivation buffer and kept at −20 °C until testing. Samples for RT-qPCR were collected with rayon swabs in 1 mL of virus transport media and kept in 4–8 °C until RNA extraction, as previously described [15].

#### 2.3.2. RT-qPCR and RNA Sequencing

The RNA extraction from the samples was performed using the NucleoSpin^®^ RNA Virus (Nagel, Germany), according to manufacturing protocols. Briefly, from 1 mL of the Virus Transport Media with the sample, 150 μL was used for RNA extraction and the remainder was stored at −80 °C for eventual repetitions or RNA sequencing. The RT-qPCR was performed using two different protocols on QuantStudio™ 3 and 5 Real-time PCR System (Applied Biosystems™, Waltham, MA, USA) and 5 Real-time PCR System (Applied Biosystems™). The main protocol followed the Charité/Berlin [16] recommendations, targeting the viral gene coding for the E protein with a reported sensibility to detect the 3.9 copies of SARS-CoV-2 genome per reaction [16]. Our internal validation disclosed 2.0 genome copies per reaction. When the result was inconclusive, we used the Center for Disease Control and Prevention (CDC) protocol [17], which targets the viral genes N1 and N2, with the sensibility to detect at least 5 copies/reaction [17]. Both protocols used the human RNAse P as the endogenous reaction control.

Thirty-five samples with Ct < 29 were selected for gene amplification, with PCR primers targeting the Spike gene, and the 1569 bp amplicons were sequenced using primers selected following the ARTIC protocol [18]. Sequences of all oligonucleotides can be found in Supplementary Table S1. The 1569 bp amplified region encodes amino acids S371, S373, S375, T376, D405, R408, K417, N440, G446, L452, S477, T478, E484, F486, Q493, G496, Q498, N501, Y505, F486, T547, A570, D614, H655, N679, P681, and S704, which have been associated with the characterization of several VOIs and the VOCs. Reverse transcription reactions and PCR amplifications were performed as previously described [19]. The assembling of Sanger contigs was performed with the GeneStudio software [Geneious Prime version 2022.0.1]. The Benchling platform [https://www.benchling.com/ (accessed on 24 August 2023)] was used for the visualization of alignments and the samples were manually genotyped via the evaluation of the modifications of interest in the sequences.

### 2.4. Expression and Purification of Nucleocapsid Recombinant Proteins DTC-N, N-Terminal Domain (Residues 47–177), and C-terminal Domain (Residues 210–419)

The codon-optimized, full-length (rDTC-N) coding region of the nucleocapsid (N) gene of SARS-CoV-2 (Genebank accession number: MT126808.1) was inserted into the pET-24a-(+) expression vector. The DNA fragments coding for the N-terminal domain (N_NTD47-177, residues 47 to 177) and C-terminal domain (N_CTD210-419, residues 210 to 419) were amplified with PCR using SARS-CoV-2 cDNA transcribed from the RNA isolated from the second Brazilian patient [20]. The primers used for PCR amplification were as follows: N_NTD47-177: 5′ AGCATAGCTAGCAATAA-TACTGCGTCTTGGTTCACCG 3′ and 5′ ATTATCGGATCCTTATCTGCTCCCTTCTGCG-TAGAAG 3′. N_CTD210-419: 5′ AGCATAGCTAGCATGGCTGGCAATGGCGG 3′ and 5′ AT-TATCGGATCCTTAGGCCTGAGTTGAGTCAGC 3′, cloned into the expression vector pET-28a. All three proteins were expressed in *Escherichia coli* strain BL21(DE3). The recombinant antigens were purified with affinity chromatography using nickel columns in an AKTA Prime plus system following the manufacturer’s instructions (GE Healthcare, Chicago, IL, USA) [21]. The eluted fractions containing the purified proteins were analyzed using SDS-PAGE, Western blot with Anti-His Tag antibody (Supplementary Figure S1). The full-length N recombinant protein was used for the mouse immunization and production of the new monoclonal antibody anti-DTC-N 1B3 (anti-DTC-N 1B3 mAb), and was applied at Ag-RDT’s control line. The proteins containing the N-terminal (N_NTD47-177) and C-terminal domains (N_CTD210-419) were used to determine the epitope localization recognized by the anti-DTC-N 1B3 mAb.

### 2.5. SARS-CoV-2 Nucleocapsid Monoclonal Antibody (mAb) Production and Characterization

The immunization protocol was followed as described before [22,23]. Briefly, 10 μg of recombinant full-length N protein (rDTC-N) was used as the antigen. The mouse with the highest antibody titer was boosted with 10 μg of rDTC-N five days prior to cell fusion. Cells from the popliteal lymph node were fused to SP2/O-Ag14 mouse myeloma cells as previously described [24]. The supernatant fluids were screened for specific antibodies via indirect ELISA, in which 100 μL of hybridoma supernatant was added to a 96-well MaxiSorp microplate (Nunc^®^, Rochester, NY, USA) previously coated with 10 μg/mL of purified rDTC-N. mAb isotyping and purification of the mAb present in the supernatant were performed as described previously [22]. The anti-DTC-N 1B3 mAb binding kinetics to recombinant DTC-N was evaluated via surface plasmon resonance (SPR) in a Cytiva Biacore T-200 system. The NTA sensor (Cytiva, Uppsala, Sweden) was equilibrated in HBS-N buffer (0.01 M Hepes pH 7.4 and 0.15 M NaCl, 25 °C) and sensitized with 50 mM NiCl_2_ for 60 s at 10 μL/min. The DTC-N protein at 1 µg/mL was captured in an NTA sensor chip (300 s, 10 µL/min). Three different sample concentrations ranging from 100 to 11.1 µg/mL (three-fold dilution) were injected sequentially (180 s association, 420 s dissociation, 30 µL/min). Between cycles, the sensor surface was regenerated with a 30 µL pulse of 0.25 M EDTA disodium. Kinetics parameters were calculated using a Langmuir 1:1 fitting model with BiaEvalutation Software 3.0. The experiment was performed in duplicate and the result was expressed as the average from the two independent assays.

#### 2.5.1. mAb Sequencing

Total RNA was extracted from 6 × 10^5^ cells producing the mAb with the RNeasy Mini Kit (QIA-GEN, Hilden, Germany), following the manufacturer’s recommendations. Reverse transcription was obtained using random hexamer primers supplied by First-Strand cDNA Synthesis (GE Healthcare, USA). The heavy and light chain variable domains’ amplification was carried out using degenerate primers [25], and the amplicons were sequenced with the SANGER method [26] to confirm the sequences as variable chains. The NGS sequencing library was prepared using 50 ng of cDNA (quantified with Qubit dsDNA BR Assay Kit–Thermo Fisher Scientific, Waltham, MA, USA) and the Nextera XT DNA Library preparation kit (Illumina, San Diego, CA, USA) using dual-index tagging, according to manufacturer’s instructions. The library’s size distribution was measured using the automated capillary electrophoresis system GelBot (Loccus, Cotia, Brazil) and the average size was used to normalize the library for the final loading concentration of 600 pM. The library was sequenced on a NextSeq 2000 P2 2 × 100 bp flow cell (Illumina, San Diego, CA, USA). The raw data were automatically converted and trimmed online at the Basespace web-based cloud (Illumina, USA). To ensure mAb had no clonal diversity, we mapped the NGS reads to the variable chains reference sequence of the cloning expression cassette, previously sequenced with SANGER using the Map to Reference tool with default parameters on Geneious Prime 2022.1.1. The alignment of the reads was manually inspected for nucleotide variation.

#### 2.5.2. Epitope Characterization and Structure Analysis

Peptide mapping was performed using PEPperPRINT (Heidelberg, Germany), according to their protocols. The N-protein sequences of SARS-CoV-2 (UniProt ID: P0DTC9), SARS-CoV (P59595), MERS-CoV (K9N4V7), HCoV-OC43 (P33469), HCoV-NL63 (Q6Q1R8), HCoV-229E (P15130), and HCoV-HKU1 (isolate 1: Q5MQC6; isolate 2: Q14EA6; isolate 5: Q0ZME3) were elongated with neutral GSGSGSG linkers at the C- and N-terminus to avoid truncated peptides. Briefly, an N-protein peptide microarray was incubated with anti-DTC-N 1B3 mAb at concentrations of 1 µg/mL, 10 µg/mL, and 100 µg/mL, followed by staining with secondary and control antibodies as well as being read out with an Innopsys InnoScan 710-IR Microarray Scanner at scanning gains of 50/10 (red/green). Microarray image analysis was undertaken with a PepSlide^®^ analyzer and summarized in the Excel file Microarray Data Mouse mAb 1B3 (PEP20225052421).xlsx.

For structure analysis, we determined the recognition pattern of anti-DTC-N 1B3 mAb between non-treated rDTC-N and treated rDTC-N (heated at 100 °C or 50 mM DTT also heated at 100 °C, both for 10 min). The mAb was also evaluated using indirect ELISA, in which MaxiSorp microplates (Thermo Fisher Scientific, Waltham, MA, USA) were coated with 10 µg/mL of rDTC-N, treated or non-treated. Phosphate-buffered saline (PBS) with bovine serum albumin (BSA) 1% was added as a blocking agent and incubated for 1 h at 37 °C. Next, the anti-DTC-N 1B3 mAb was diluted (log2) in an initial concentration of 7.15 µg/mL, followed by incubation with goat anti-mouse IgG conjugated with horseradish peroxidase (Sigma-Aldrich, St. Louis, MO, USA) diluted 1:5000 in PBS-BSA 0.1% solution. Reactions were developed with 0.5 mg/mL O-phenylenediamine (OPD; Sigma Aldrich Co., St. Louis, MO, USA) plus 0.5-μL/mL hydrogen peroxide in 0.05 M citrate-phosphate buffer, pH 5.0, in the dark, at room temperature. The reactions were interrupted after 15 min by the addition of 50 μL of 1 M HCl. The absorbance was measured at 492 nm in a Multiskan EX ELISA reader (Labsystems, Milford, MA, USA). At each step, the volume added was 100 μL/well, except in the washing and blocking steps, when the volume used was 200 μL/well. Between incubation periods, the plates were washed three times with PBS-Tween 0.05%. All experiments were carried out in technical duplicates, and the results corresponded to three independent experiments (biological replicates). The nitrocellulose membranes (GE-Healthcare, Freiburg, Germany) containing the transferred proteins DTC-N (SARS-CoV-2), N-terminus protein N (SARS-CoV-2), C-terminus protein N (SARS-CoV-2), DENV-2 NS1 recombinant protein and full protein N (SARS-CoV-2) from 12% polyacrylamide gel electrophoresis containing sodium dodecyl sulfate (SDS-PAGE) were blocked with BSA 1% for 1 h at 37 °C and then they were tested with an anti-DTC-N 1B3 mAb (1:100) at a concentration of 870 µg/mL. Next, the membranes were incubated with goat anti-mouse IgG conjugated with peroxidase (1:5000). The reactive protein bands were identified with 10 mg DAB in 15 mL Tris-buffered saline plus 12 µL H_2_O_2_ (30%).

### 2.6. SARS-CoV-2 Samples

The SARS-CoV-2 viral strains used in this study were from the lineage B (isolate BRA/SP02/2020), Delta (EPI_ISL_2965577), and Omicron (EPI_ISL_7699344) variants. Viral stocks were propagated in Vero E6 cells (ATCC CRL-1586) at 37 °C with 5% CO_2_ and observed for cytopathic effects (CPE) daily up to 72 h. Viruses were titrated in Vero E6 cells using a plaque forming units (PFU) assay [20]. Viral aliquots were kept at −80 °C until further use. The identity of all samples was confirmed by genome sequencing. Before use, samples were heated at 60 °C for 1 h to inactivate the virus. Parallel analyses of the diluted stock viruses were performed using RT-qPCR and the developed Ag-RDT. The results obtained were compared with the purpose of certifying the ability of the Ag-RDT to specifically detect the original SARS-CoV-2 virus, as well as its variants.

### 2.7. Antigen Rapid Diagnostic Test (Ag-RDT)

The Ag-RDT developed was based on the lateral flow immunochromatographic principle. Several membranes were superposed, each one containing a specific reagent. The test and control lines were sprayed and immobilized on the nitrocellulose membrane using the Continuous Dispenser HGS101 (Autokun^®^, Hangzhou, China). The antibodies were conjugated to colloidal gold nanoparticles [27] and dispensed onto a glass fiber. The glass fiber was also used on the sample pad. A cellulose fiber was used to absorb all the reagents at the opposite end of the test membranes. The superposed membranes’ strips were placed on a plastic cassette, which was the dispositive where the test was performed. In the presence of SARS-CoV-2, the virus N protein reacted with the monoclonal antibody in the conjugate pad and as the complex flowed through the nitrocellulose membrane, it was captured by a second monoclonal antibody dispensed on the test line. The results were visually read 20 min after sample application, and any color intensity formed in the test line was considered positive. In the absence of SARS-CoV-2, no reactivity was presented at the test line. The remaining conjugate, bound to the control line, resulted in a color change that was required to validate the test.

#### 2.7.1. Inactivation Buffer

The inactivation buffer used to collect the sample for the Ag-RDT was designed to lyse the cells and expose the viral antigens, as well as to maintain the structure of the viral protein. It contained Tris-NaCl buffer (pH 8.5) and Tergitol^®^ NP-40 as a lysis agent. Nasal swab samples were collected in vials containing 0.3 mL of buffer.

#### 2.7.2. Prototype 1

Initially, we used a pair of commercial monoclonal antibodies anti-N (mAb A and mAb B) from Fapon Biotech^®^ (Dongguan, China), to prototype an Ag-RDT (prototype 1). By using this pair of mAb previously tested in the prototype, we were able to set up all the other components and variables that may interfere with an Ag-RDT performance. Prototype 1 was then used as a proof of concept for the correct assembly of all test components and reagents. The mAb A was immobilized on the nitrocellulose membrane as the test line in a concentration varying between 0.2 and 4.0 mg/mL. The recombinant protein N was dispensed as the control line in the same concentration range as the test line. The mAb B was used as the conjugate. In this way, the mAb B was mixed with colloidal gold nanoparticles of 20 nm, incubated at room temperature for 5 min, and blocked with BSA (Sigma-Aldrich^®^) for the same time. After this process, the solution was centrifuged at 8 °C, the supernatant was discarded, and the pellet was resuspended in a storage buffer, dispensed on glass fiber, and dried at room temperature and <40% humidity. The strips were superposed, cut, and placed in plastic cassettes.

#### 2.7.3. Prototype 2

After prototype 1 had been set up and tested, we gradually replaced one of the commercial mAb with the newly developed anti-DTC-N 1B3 mAb (mAb C), generating prototype 2. A mixture of mAb A and mAb C was immobilized on the nitrocellulose membrane as the test line in a concentration varying between 0.2 and 4.0 mg/mL. All the remaining conditions used for prototype 1 were maintained for this second prototype.

#### 2.7.4. Prototype 3

In prototype 3, the commercial mAb B was completely replaced with mAb C, as the conjugate, using colloidal gold nanoparticles of 40 nm. All other remaining conditions of prototype 1 were maintained.

### 2.8. Assaying Ag-RDT Prototypes with SARS-CoV-2 Virus

Before starting the tests, all reagents and samples were brought to room temperature. The samples were homogenized, and 120 µL of samples were applied to the sample cavity of the cassette. All three prototypes were initially evaluated using dilutions of the SARS-CoV-2 virus collected in DMEM medium and inactivated by heat, as described above. Virus stocks’ dilutions (1:2 up to 1:1024) were prepared using the inactivation buffer, which was used as negative control.

### 2.9. Assaying Ag-RDT Prototypes with Patients’ Samples for Validation

As previously mentioned, prototype 1 was used as a proof of concept for the correct assembly of all test components and reagents and was assayed using 17 swab samples of patients, 10 of which were negative and 7 positive in RT-qPCR. For the validation of prototypes 2 and 3, 2 different sets of 120 positive and 60 negative samples of patients from the Guaranésia cohort were assayed on each prototype, and the results were compared to the results of RT-qPCR. Two sets of samples were required for the evaluation of each prototype, due to the limitation of the sample’s volume. For each test, 120 µL of samples were added and the results were read after 20 min.

### 2.10. Stability Assay

The stability assay was performed by the aggression of prototypes at 45 °C, to estimate the real-time stability of the Ag-RDT [28]. The tests were packed in sachets containing silica gel and sealed before being placed at 45 °C. They were evaluated at day 0 (before being placed in the incubator) and every 7 days after that. For the evaluation, 4 cassettes were removed from the stove and evaluated with serial dilutions of heat-inactivated SARS-CoV-2 virus stocks and with an inactivation buffer (negative control). For each test, 120 μL of samples were added and the results were read after 20 min. For the determination of real-time stability, samples of Ag-RDT prototype 3 were maintained under standard storage conditions, at room temperature and under humidity control. The evaluation procedure was performed as described above for over 14 months.

### 2.11. Statistical Analysis

Descriptive statistics were used to analyze the data. Sensitivity was defined as the proportion of SARS-CoV-2 positive patients correctly identified by the Ag-RDT that was also positive by RT-qPCR. Specificity was defined as the proportion of samples correctly identified as negative by Ag-RDT and also categorized as negative by RT-qPCR. Sensitivity was evaluated globally and according to the Ct value for the E gene or the N gene using different cutoffs (Ct ≤ 25; 25 ≤ Ct ≤ 30 and Ct > 30) and the days post onset of symptoms (0–3 days; 4–7 days; >7 days). Accuracy was calculated by dividing the sum of the true positives and true negatives by the total number of samples analyzed. The concordance between RT-qPCR and Ag-RDT was calculated using the Kappa (k) index, according to Cohen [29]. Statistical analysis was carried out using MedCalc version 20.118 (MedCalc Software) and the GraphPad Prism 8.0.1 software. The 95% confidence intervals (CI) for sensitivity, specificity, and accuracy were calculated using the Clopper–Pearson method. The Mann–Whitney test was used to estimate statistical differences between pairs of tests. Differences were considered statistically significant with the *p*-value < 0.05.

Statistical analyses of data obtained for the characterization of anti-DTC-N 1B3 mAb were performed with two-way variance analysis followed by the ANOVA post-test. (** *p* = 0.0018). Differences between means of the ELISA optical density of reactivity with the intact protein and treated fractions were analyzed using an unpaired Student’s *t*-test; *** *p* = 0.0009 compared to DTT-treated protein and * *p* = 0.01 compared to heat-treated protein.

## 3. Results

### 3.1. Population Characteristics

From 1 December 2021 to 8 February 2022, 939 samples were collected in the city of Guaranésia, Minas Gerais, for simultaneous analysis using RT-qPCR and Ag-RDT. The distribution of positive and negative samples and their corresponding Ct values are shown in Supplementary Figure S3. Among the 939 samples evaluated, 22.68% (213/939) were negative for SARS-CoV-2 on RT-qPCR analysis, 76.25% (716/939) were positive, based on the detection of the gene E sequence, and 1.06% (10/939) showed an inconclusive result. From the positive samples, 64.4% (461/716) showed Ct values ≤ 25; 11.59% (83/716) showed Ct values between 25 and 30; and 24.0% (172/716) had a Ct value > 30. For the evaluation of the Ag-RDT prototype 2, 180 samples characterized with RT-qPCR were selected: 60 (33.3%) negative samples and 120 (66.7%) positive samples (65.8% with Ct values ≤ 25; 15.0% with 25 < Ct value ≤ 30 and 19.2% with Ct value > 30). For the evaluation of the Ag-RDT prototype 3, an additional 180 samples also characterized with RT-qPCR were selected: 60 (33.3%) negative samples and 120 (66.7%) positive samples (70.0% with Ct values ≤ 25; 12.5% with Ct value between 25 and 30 and 17.5% with Ct value > 30). The positive samples were selected in a way to approximate the proportion of Ct values detected in the population.

For the follow-up study, 112 positive samples detected using RT-qPCR were collected sequentially after the onset of symptoms from 38 patients living in the city of Belo Horizonte, from 23 December 2021 to 15 February 2022. According to the day of onset of symptoms, 22.3% (25/112) of the samples were collected between 0 and 3 days, 40.2% (45/112) between 4 and 7 days, and 37.5% (42/112) were collected from the 8th until the 17th day of the symptom onset. The Ct means found in the RT-qPCR for each group were 21.5, 25.2, and 30.8, respectively.

DNA sequencing, performed in 35 samples from Guaranésia and Belo Horizonte cities, indicated the predominance of the Omicron BA.1 variant of SARS-CoV-2, which was detected in 100% of the samples analyzed.

### 3.2. SARS-CoV-2 Nucleocapsid mAb (Anti-DTC-N 1B3 mAb) Functional Characterization

**Figure 1:**
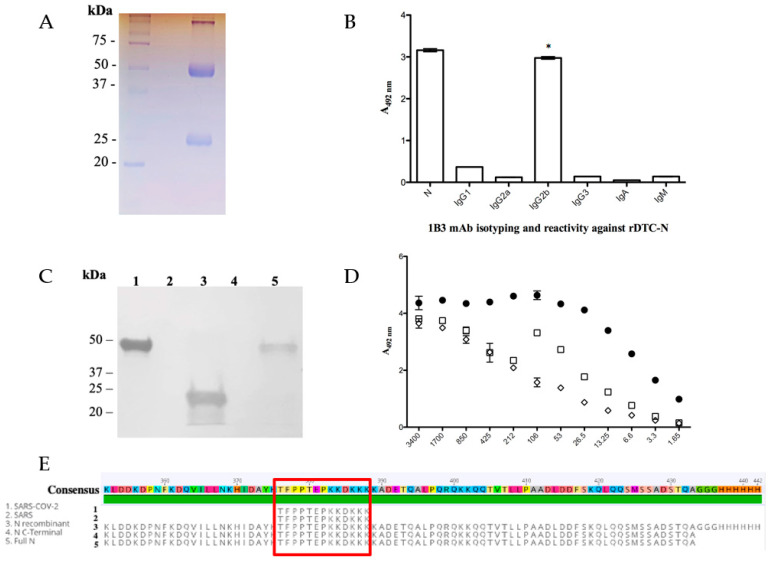
Anti-DTC-N 1B3 mAb purification and characterization. (**A**) The 12% SDS/PAGE anti-DTC-N 1B3 mAb profile stained with Coomassie Blue after the G protein affinity column purification. (**B**) Anti-DTC-N 1B3 mAb isotyping analysis. C96 MaxiSorp ELISA microtiter plates coated with 10 µg/mL of recombinant rDTC-N or 1 µg/mL of IgG1, 2a, 2b, IgG3, IgA, and IgM, incubated with 100 µL supernatants of anti-DTC-N 1B3 mAb and with rat anti-mouse kappa conjugated with horseradish (1:1000). The asterisk (*) indicates statistically significant differences as compared to all the other isotypes of IgG, and IgA and IgM subclasses (*p* ≤ 0.0001). (**C**) Immunobloting analysis. Nitrocellulose membrane containing 10 µg of (1) DTC-N (SARS-CoV-2); (2) N-terminus protein N (SARS-CoV-2); (3) C-terminus protein N (SARS-CoV-2); (4) DENV-2 NS1 recombinant protein; (5) full-length control protein N (SARS-CoV-2). Membrane was probed with the anti-DTC-N 1B3 mAb (1:100) at a concentration of 870 µg/mL and goat anti-mouse IgG conjugated with peroxidase (1:5000). (**D**) Anti-DTC-N 1B3 recognition patter. The 10 µg/mL rDTC-N, heated-treated [100 °C for 10 min (□), DTT-treated (◇), or intact (●) were used as solid phase-bound antigens. The anti-DTC-N 1B3 mAb was serially diluted (log2) from an initial concentration of 0.007 μg/mL. (**E**) Alignments of the anti-DTC-N 1B3 mAb epitope with N protein sequences. The epitope sequence recognized by anti-DTC-N 1B3 mAb was aligned with protein N partial sequences, comprised between amino acids 352 and 434 from SARS-CoV-2, SARS, and with sequences obtained for the full-length N and N-terminal recombinant proteins.

After fusion, only one secretory hybridoma was generated, named 1B3. The clone was expanded, supernatants were collected and the mAb was purified (Figure 1A). The mAb-protein interaction association and dissociation rates (mean ± SD, *n* = 2) were measured using surface plasmon resonance (SPR); rates of (7.2 ± 0.5) × 10^3^ M^−1^ s^−1^ and (3.27 ± 0.01) × 10^−4^ s^−1^, respectively, were found with a corresponding binding affinity *K*_D_ of (4.5 ± 0.3) × 10^−8^ M. To determine the anti-DTC-N 1B3 isotype, antibodies that recognized specifically IgG1, 2a, 2b, IgG3 (*p* ≤ 0.0001), and IgA and IgM subclasses were used in ELISA. Significant reactivity (*p* ≤ 0.0001) with the N protein was observed only for IgG2b, when compared to all the other isotypes (Figure 1B). The location of the epitope recognized by the anti-DTC-N 1B3 on the N protein was confirmed via immunoblotting (Figure 1C). This analysis revealed that the anti-DTC-N 1B3 mAb recognizes not only the full-length N recombinant protein, but also its C-terminal domain. No reactivity was detected in the N-terminus region. The recognition pattern of the anti-DTC-N 1B3 mAb was then evaluated with ELISA using either non-treated rDTC-N or treated rDTC-N (heated at 100 °C or 50 mM DTT also heated at 100 °C, both for 10 min). The results indicated that the recognized epitope was at least partially represented by conformational structures, since reactivity was affected by treatments that may affect the N protein structure (Figure 1D). The epitope mapping resulted in the recognition of peptides with the consensus motif TFPPTEPKKDKKK of SARS-CoV-2, and SARS-CoV (Figure 1E), which aligned with the C-terminus sequence of the protein and was not mutated in the Omicron variant (Supplementary Figure S2). Anti-DTC-N 1B3 mAb CDRs domains were confirmed using the SANGER method after random primers’ detection and the NGS sequencing library (Supplementary Figure S1).

### 3.3. Ag-RDT Validation

#### 3.3.1. Prototype 1 Performance

**Figure 2:**
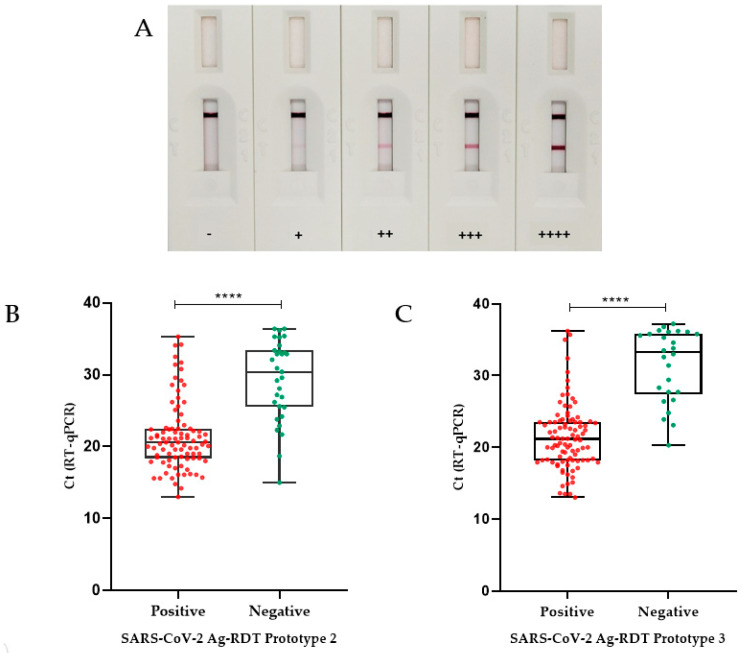
Validation of Ag-RDT prototypes based on RT-qPCR. (**A**) Illustrative figures of Ag-RDT results. Figures show tests with different intensities of reaction as detected by the Ag-RDT. According to the signal strength of the positive samples on the test line (T), the results were classified as + (low intensity), ++ (moderate to low intensity), +++ (moderate to high intensity), and ++++ (high intensity). In the absence of the appearance of a test line (T), the sample was considered negative (−). (**B**) Box plot analysis of RT-qPCR and Ag-RDT prototype 2. RT-qPCR Ct values (for positive samples only) as compared with positive and negative results obtained with prototype 2 Ag-RDT. The median Ct value found for the false-negative samples on SARS-CoV-2 Ag-RDT (30.4) was significantly different from the median Ct value found for the positive samples on SARS-CoV-2 Ag-RDT (20.6). (**C**) Box plot analysis of RT-qPCR Ct value (positive only) compared with prototype 3 Ag-RDT results. The median Ct value found for the false-negative samples on SARS-CoV-2 Ag-RDT (33.2) was significantly different from the median Ct value found for the positive samples on SARS-CoV-2 Ag-RDT (21.1) (*p* < 0.0001 was indicated by ****). Boxplot analysis (2B and 2D) was performed by applying the Mann–Whitney test, using the GraphPad Prism 8.0.1 software. The line across the box is the median. The whiskers represent all points showing minimum to maximum quartiles.

Prototype 1, used only as a proof of concept, was initially tested against the SARS-CoV2 variants’ culture stocks (Wuhan, Alpha, Gamma, Delta, P2, and Omicron). Positive results were detected in samples from all tested SARS-CoV-2 cultured stocks in dilutions of up to 1:1024, with high signal intensity, regardless of the variant. This prototype detected five out of seven positive patient samples with Ct values between 19.1 and 26.4, whereas the two undetected samples had Ct values of 29.5 and 32.7. Nonspecific reactions were not observed in any of the 10 negative samples evaluated. Color intensities of the reactions for this prototype are shown in Figure 2A.

#### 3.3.2. Prototype 2 Performance

Sensitivity results (95% CI) for prototype 2 were extracted according to the Ct of the samples obtained using the RT-qPCR technique. The sensitivity values obtained for samples with Ct ≤ 25 was 91.1% (CI 82.6–96.4%); for samples with Ct between 25 and 30 it was 55.6% (CI 30.8–78.5%); and for samples with Ct > 30 it was 30.4% (CI 13.2–52.9%). The overall sensitivity of this Ag-RDT prototype was 74.2% (CI 65.4–81.7%). The specificity of the test was 90% (CI 79.5–96.2%) and the overall accuracy found was 79.4% (CI 72.8–85.1%) (Table 1). The median Ct found for the false-negative samples (30.4) was significantly different (*p* < 0.0001) from the median value determined for the positive ones (20.6), according to the Mann–Whitney test (Figure 2B).

The agreement between Ag-RDT and RT-qPCR results for all samples corresponded to a moderate Kappa index (agreement = 79.4%, k = 0.579). When considering only samples with high viral loads (Ct ≤ 25), an excellent concordance was obtained (agreement = 90.6%, k = 0.811).

Prototype 2 was stable for 45 days at 45 °C, which corresponded to approximately 12 months at room temperature.

#### 3.3.3. Prototype 3 Performance

To improve Ag-RDT specificity and to reduce the dependency on commercial antibodies, we developed prototype 3 by replacing mAb B with mAb C (anti-DTC-N 1B3 mAb). As shown for prototype 2, prototype 3 was also able to detect all analyzed SARS-CoV-2 variants—Wuhan, Alpha, Gamma, Delta, P2, and Omicron. The sensitivity (95% CI) for prototype 3 was also categorized according to the Ct of the samples obtained with the RT-qPCR. The sensitivity obtained for samples with Ct ≤ 25 was 95.2% (CI 88.2–98.7%); it was 60.0% (CI 32.3–83.6%) for samples with Ct between 25 and 30, and 23.8% (CI 8.2–47.2%) for samples with Ct > 30. The specificity of the test was 98.3% (CI 91.1–99.9%) and the overall accuracy found was 85.0% (CI 78.9–89.9%) (Table 1). The median Ct found in the false-negative samples (33.2) was significantly different (*p* < 0.0001) from the median value of the positive ones (21.1), as detected using the Mann–Whitney test (Figure 2C). Prototype 3 has displayed a real-time stability of 14 months, so far.

### 3.4. Ag-RDT Follow-Up Study

The sensitivity of Ag-RDT tests may vary during the progress of the SARS-CoV-2 infection, after the onset of symptoms [30]. Therefore, we performed sequential sampling for patients from the Belo Horizonte cohort, starting from the day of PCR diagnosis and symptoms onset. This follow-up study indicated that whenperformed within the first three days after the onset of symptoms, when patients displayed a mean Ct of 21.5, the Ag-RDT displayed a 100% (CI 86.3–100.0%) sensitivity. Between 4 and 7 days after symptom onset, the sensitivity of the Ag-RDT was reduced to 64.4% (CI 48.8–78.1%), whereas the percentage of positive results obtained from the eighth day post symptoms onset was further reduced to 42.8% (CI 27.7–59.0%) (Table 2). As noted before, the sensitivity observed between the days after symptoms onset correlated with the viral load, i.e., it decreased with the increase in the mean Ct of the samples.

A summary of the prototypes’ performances compared to some commercial kits is presented in Table 3. The reported sensitivity of commercial kits varies from 92.9% to 100% for samples with Ct ≤ 25, while specificity varies from 83.3% to 100%.

## 4. Discussion

After more than 3 years of the COVID-19 pandemic, SARS-CoV-2 Ag-RDT has become an important alternative for diagnosis, patient follow-up and to improve the efficacy of disease control. While the effective implementation of Ag-RDTs requires constant surveillance of their analytical performance, as new SARS-CoV-2 variants may emerge, new reagents and test availability may ensure that a loss of sensitivity of Ag-RDTs will be specifically overcome.

Here, we report a complete engineering process for developing a SARS-CoV-2 Ag-RDT, including the production of N recombinant protein as well as the production and characterization of a new mAb (anti-DTC-N 1B3 mAb) that is specific for this protein. So far, more than 30 mutations, some of them very frequent, have been reported among the variants of SARS-CoV-2. In contrast, only four main mutations are present in N protein [38,39]. Therefore, the use of mAbs directed to different and conserved epitopes of the N protein may improve sensitivity, overcoming losses caused by the high mutation rates of Spike as a target antigen. Indeed, anti-N mAbs have been successfully used for the development of commercially available Ag-RDT tests [10,12,40], as well as in other sensitive assays [41]. In addition to the high mutation rate, the Spike protein has several sites of post-transcriptional modifications and usually requires eukaryotic expression systems for its expression as a recombinant protein in a correct conformation [42]. In contrast, the N protein was easily expressed in prokaryotic cells, which is convenient for the generation of monoclonal antibodies and its use as a reagent in RDT.

To prototype the Ag-RDT, initially we used a pair of antibodies for the detection of the virus N antigen, which were commercially available. Using these mAbs, all the reagents, membranes and detection conditions were tested. Then, the commercial mAb was replaced with the new mAb anti-DTC-N 1B3. The epitope mapping of the anti-DTC-N 1B3 mAb revealed a sequence present in the C-terminus of the N protein that was conserved in all main VOCs of SARS-CoV-2 analyzed. To address this question experimentally, we tested the performance of the Ag-RDT prototypes with viral culture stocks corresponding to the main VOCs that circulated globally. In agreement, the Ag-RDT prototypes were able to detect all tested variants of SARS-CoV-2 and showed a performance similar to commercial tests [43].

The performance of the Ag-RDT prototypes was also challenged in a real-time validation test with samples collected from patients during an outbreak of the Omicron variant in Brazil. According to WHO, Ag-RDTs should meet a minimum performance requirements of ≥80% sensitivity and ≥97% specificity [7]. Since the specificity of prototype 2 was 90% and, therefore, beneath the value recommended by WHO, an improved prototype was developed by replacing the commercial mAb B with the anti-DTC-N 1B3 mAb. Prototype 3 showed 98.3% specificity and 95.2% sensitivity when tested with samples with Ct ≤ 25, meeting WHO’s criteria. This performance was also similar to those found for tests currently available on the market. The distribution of the Ct values of true positive and false negative samples and the concordance index of Ag-RDT with RT-qPCR were also comparable to those reported for commercial SARS-CoV-2 antigen tests [30,32], indicating the potential of the Ag-RDT developed here as a tool for COVID-19 diagnosis. Although a sensitivity of 100% has been reported for some commercial tests, direct comparisons of their performance with our data may be compromised given the differences in VOCs prevalence, the vaccination status of the populations and the assay conditions for comparison with RT-qPCR [44]. In agreement, Nora et al. (2022) reported the evaluation of 10 commercial kits’ on-field conditions and found, for some of them, important differences on their performance when compared to previously reported data [36,37].

The follow-up study aimed at evaluating the correlation between the dynamics of viral loads, after symptoms onset and the sensitivity of the Ag-RDT, since this may provide additional information on the performance of the Ag-RDT in patients with confirmed diagnosis at different time points, either with high or low viral loads. As observed in other studies [30,44], and following the dynamics of viral infection, the Ct of the samples increased after the symptoms onset, reaching the detection limit of the assay, evidencing a constant decrease in the viral loads. In our study, this decrease was more pronounced after 4 days past symptoms onset, when patients presented a mean Ct > 25.

RT-qPCR is considered the gold standard for COVID-19 diagnosis, and thus used to evaluate the performance of Ag-RDTs. Nonetheless, some aspects may be considered for results’ interpretations when comparing these tests, given that targets and conditions for these assays are quite different. In the present study, samples for Ag-RDT and RT-qPCR were collected concomitantly for each patient, but from different nostrils and maintained in different buffers. These different conditions may have introduced a bias in the viral load between the two samples. Another aspect is the fact that it is still unclear whether the persistent detection of SARS-CoV-2 RNA in routine nasopharyngeal swabs of COVID-19 patients represents replicating virus or simply viral nucleic acid in cell debris [45]. There is no clear evidence for a correlation between RNA viral load and infectivity and transmissibility, i.e., whether RT-qPCR results in patients with Ct > 25 indicate the presence of intact viral particles or reminiscent sub-genomic fragments [46]. This correlation is even more complicated when extrapolated for Ag-RDT, since the targets for RT-qPCR and Ag-RDT are different. Despite the lower sensitivity when compared to molecular testing, it has been argued that antigen tests may be a better indicator of viral infectivity. Nevertheless, there is a significant consensus that, in the presence of symptoms, a negative Ag-RDT test should be validated by a RT-qPCR test [47].

It has been reported that vaccination affects the duration of symptoms and the viral loads measured with RT-qPCR [48]. There is also evidence that SARS-CoV-2 T cell responses, besides neutralizing antibodies induced by either natural infection or vaccines, limit the disease severity by providing a rapid viral clearance [49,50,51,52]. The developed prototypes were validated during the outbreak of the Omicron variant in the city of Guaranésia, Minas Gerais, Brazil, after this population had already reached a significant level of vaccination coverage. The sensitivity of prototypes dropped among patients from this population with Ct values higher than 25. Therefore, the high rate of vaccination in Guaranésia’s population may explain the decrease in the sensitivity values for the Ag-RDT prototypes tested in patients with Ct > 25.

Whilst our study aimed at contributing with new reagents and valuable insights into the detection and characterization of SARS-CoV-2 and its variants, some limitations are noteworthy, including testing on a limited sample size and the evaluation of a specific population, from a single region, in a specific period of time. Further independent validation studies could enhance the robustness of our findings and confirm the diagnostic accuracy of the Ag-RDT in more diverse settings. Whereas our assay exhibited high sensitivity, it is important to acknowledge that false negatives might occur, especially in cases of low viral loads. Optimizing sensitivity for varying viral loads could enhance its effectiveness. The potential for bias in sample collection, even with rigorous protocols, could impact the representativeness of our study population. Strategies to minimize selection bias during sample collection would boost the validity of our results. While the developed prototypes showed specificity for SARS-CoV-2, potential cross-reactivity with other related pathogens was not exhaustively investigated. Moreover, the potential for detecting emerging variants, such as those associated with VOCs, warrants the continuous surveillance and adaptation of the diagnostic test. In addition, further studies evaluating the stability of our assay components over extended periods would provide confidence in its reliability. Finally, some of these limitations may be related to the fact that the prototypes were produced in a laboratory setting. Industrial prototypes may be further optimized, allowing adjustments to improve the performance and additional validation assays.

## 5. Conclusions

Results of this study indicated that, using a new anti-N mAb, the Ag-RDT developed displayed high sensitivity and specificity, regardless of the most significant VOCs circulating worldwide and the immune status of the population, and may, therefore, constitute an alternative tool for improving point-of-care diagnosis and for epidemiological and follow-up studies of COVID-19.

## Figures and Tables

**Table 1:** Prototype 2 and 3’s performances according to RT-qPCR results.

Prototype 2						
RT-qPCR Positive Samples	Ct	Ag-RDT Positive	Ag-RDT Negative	Sensitivity (95% CI)	Specificity (95% CI)	Accuracy (95% CI)
79	Ct ≤ 25	72	7	91.1% (82.6–96.4%)	90% (79.5–96.2%) 54/60	90.6% (83.5–94.9%)
18	25 < Ct ≤ 30	10	8	55.6% (30.8–78.5%)		82.1% (71.7–89.8%)
23	Ct > 30	7	16	30.4% (13.2–52.9%)		71.2% (62.7–82.6%)
120	Total	89	31	74.2% (65.4–81.7%)		79.4% (72.8–85.1%)
**Prototype 3**						
**RT-qPCR** **positive sample**	**Ct**	**Ag-RDT positive**	**Ag-RDT negative**	**Sensitivity** **(95% CI)**	**Specificity** **(95% CI)**	**Accuracy** **(95% CI)**
84	Ct ≤ 25	80	4	95.2% (88.2–98.7%)	98.3% (91.1–99.9%) 59/60	96.5% (92.1–98.8%)
15	25 < Ct ≤ 30	9	6	60.0% (32.3–83.6%)		90.7% (81.7–96.2%)
21	Ct > 30	5	16	23.8% (8.2–47.2%)		79.0% (68.5–87.3%)
120	Total	94	26	78.3% (69.9–85.3%)		85.0% (78.9–89.9%)

95% CI: calculated with the Clopper–Pearson method using MedCalc Software.

**Table 2:** Diagnostic performance of the developed Ag-RDT (prototype 2), according to the day of symptoms onset.

Days of Symptoms Onset	RT-qPCR Positive Samples	Mean Ct	Ag-RDT Positive Samples	Sensitivity (95% CI)
0 to 3	25	21.5	25	100.0% (86.3–100.0%)
4 to 7	45	25.2	29	64.4% (48.9–78.1%)
>7	42	30.8	18	42.8% (27.7–59.0%)

95% CI: calculated with the Clopper–Pearson method using MedCalc Software.

**Table 3:** Summary of the prototypes’ performances compared to commercial kits.

Author	Country	Samples (*n*)		Commercial Kit	Sensitivity		Specificity
		+	−		Ct ≤ 25	Overall	
Rastawicki, 2021 [31]	Poland	95	46	PCL COVID-19 Ag Rapid FIA (ROK)	92.9%	38.9%	83.3%
Pérez-García, 2021 [32]	Spain	186	170	Panbio COVID-19 Ag Rapid Test Abbott (USA)	98.9%	60.0%	100%
				SD Biosensor Ag (ROK)	97.4%	66.5%	97.3%
Blairon, 2021 [33]	Belgium	150	49	Coronavirus Ag Rapid Test Cassette Bio-Rad (USA)	97.1%	60.0%	100%
				GSD NovaGen SARS-CoV-2 Antigen Rapid Test (China)	95.7%	59.3%	85.7%
				Aegle Coronavirus Ag Rapid Test Cassette LumiraDx (UK)	97.1%	61.1%	100%
Sood, 2021 [34]	USA	226	548	BinaxNOW™ Abbott (USA)	93.8%	56.2%	98.4%
Choudhary, 2022 [35]	India	129	627	SD Biosensor, Inc. (ROK)	NR	55.0%	99.2%
Nóra, 2022 [36]	Hungary	40	58	GenBody COVID-19 Ag (ROK)	93.8%	62.0%	86.4%
Wegrzynska, 2023 [37]	Poland	103	301	GenBody COVID-19 Ag (ROK)	100%	97.1%	100%
Prototypes	Brazil	120	60	SARS-CoV-2 Ag RDT Prototype 2 (Brazil)	92.0%	74.1%	90.0%
		120	60	SARS-CoV-2 Ag RDT Prototype 3 (Brazil)	95.2%	78.3%	98.3%

NR: Not reported; +: Positive; −: Negative.

## Data Availability

Not applicable.

## Supplementary Materials

The following supporting information can be downloaded at: https://www.mdpi.com/article/10.3390/microorganisms11102422/s1, Table S1. Primers used for PCR amplification and Sanger sequencing. In the table are present the identifiers and sequences of the primers (Primer ID, Sequence (5′–3′). The nCoV-2019_75_LEFT, nCoV-2019_77_RIGHT and CTR1 primers were used for the obtention of 1569 bp. Figure S1. Anti-HisTag immunoblotting of purified Nucleocapsid fragments. A. Full-length (N_Full1-419); B. N-terminal domain (N_NTD47-177); C. C-terminal domain (N_CTD210-419); D. Precision Plus Protein Kaleidoscope Standards (Bio-Rad). Figure S2. Protein N sequence across SARS-CoV-2 variants of concern (VOC) Alpha (B.1.1.7), Beta (B.1.351), Gamma (P.1), Delta (B.1.617.2) and Omicron (B.1.1.529) compared with Wuhan (Hu) sequence as reference. Yellow squares represent most mutagenic sites. The red region represents the conservate epitope among SARS-CoV-2 lineages, which is recognized by monoclonal antibody in ag-RDT. Sequences aligned by multalin (http://multalin.toulouse.inra.fr/multalin/ accessed on 27 September 2022). Image adapted from Biorender (http://biorender.com). Figure S3. Cohort samples’ characterization using RT-qPCR. [A] Samples collected from patients of the cohort of Guaranésia City were categorized according to the Cycle threshold (Ct) values detected in positive samples and arranged in a sectorized percentage graph. Samples from this cohort were selected to evaluate the performance of prototype 1, as proof of concept, and to validate prototypes 2 and 3, aiming to maintain the Cts distribution in the same or as close as possible to the Cts proportions of the original population. [B] The follow-up study samples were longitudinally grouped as 0–3 days, 4–7 days, or more than 7 days after symptoms onset, respectively, and characterized using RT-qPCR and Ag-RDT.
