# Supplementary material for: SARS-CoV-2 Rapid Antigen Test Based on a New Anti-Nucleocapsid Protein Monoclonal Antibody: Development and Real-Time Validation
Source: Microorganisms. 2023 Sep 28;11(10):2422. doi: 10.3390/microorganisms11102422 (PMC10608853; doi:10.3390/microorganisms11102422)
Supplement: Supplementary file 1 [file microorganisms-11-02422-s001.zip › microorganisms-2529537-supplementary.pdf]

Supplementary Materials

Table S1

Primer ID	Sequence (5' - 3')
nCoV-2019_75_LEFT	AGAGTCCAACCAACAGAATCTATTGT
nCoV-2019_77_RIGHT	CAGCCCCTATTAAACAGCCTGC
CTR1	CATATTGCAACAAAAGATTGCTGCATT

Figure S1

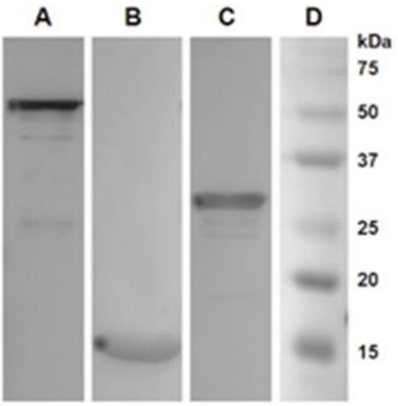

Figure S2

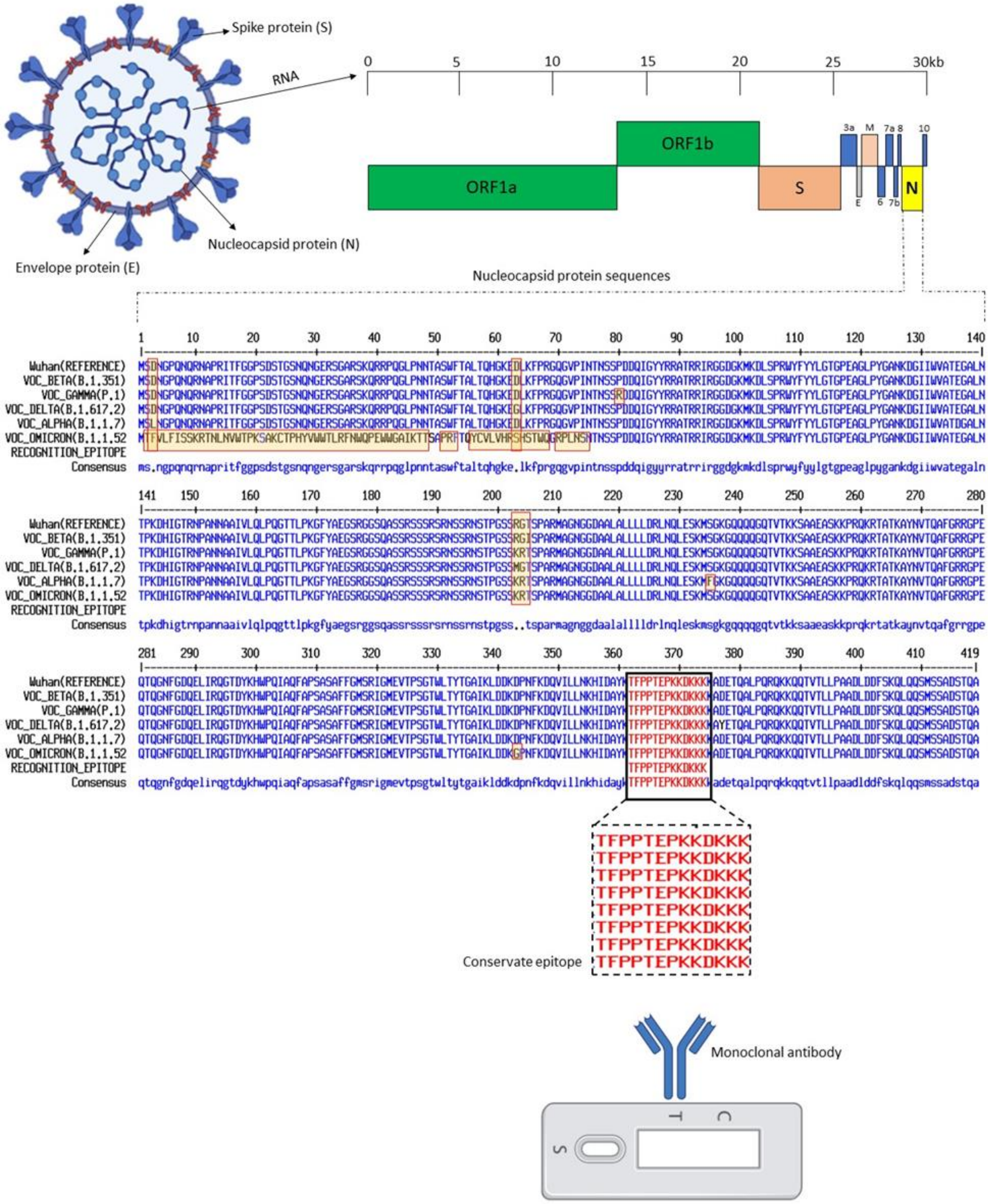

Spike protein (S)
RNA
Nucleocapsid protein (N)
Envelope protein (E)
0
5
10
15
20
25
30kb
ORF1a
ORF1b
S
3a
M
7a
8
10
E
6
7b
N
Nucleocapsid protein sequences
Wuhan(REFERENCE)
VOC_BETA(B.1.351)
VOC_GAMMA(P.1)
VOC_DELTA(B.1.617.2)
VOC_ALPHA(B.1.1.7)
VOC_OMICRON(B.1.1.52
RECOGNITION_EPITOPE
Consensus
TFPPTEPKKDKKK
Conservate epitope
Monoclonal antibody
S
T
C

Figure S3

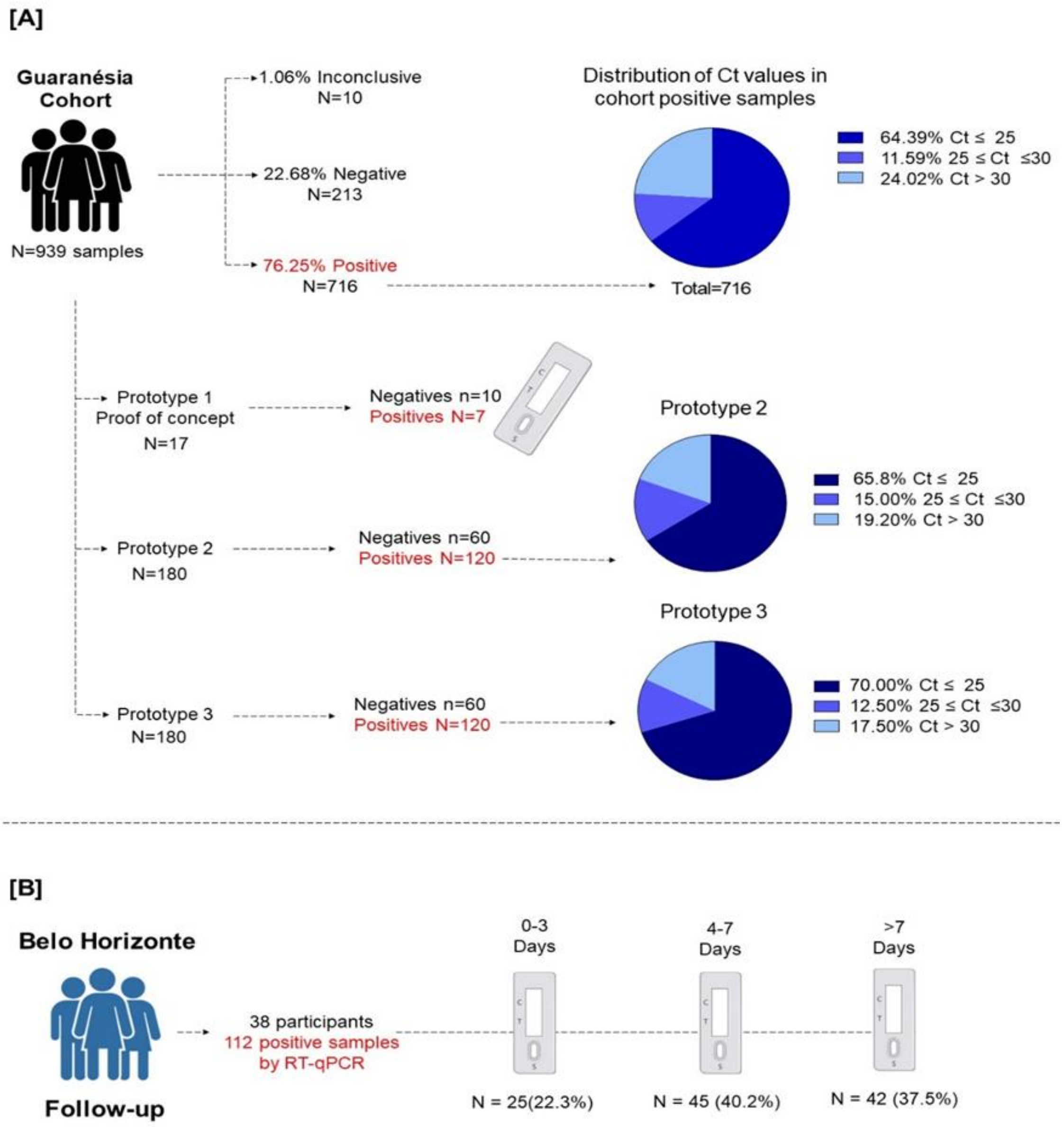
[A]
Guaranésia Cohort
N=939 samples
1.06% Inconclusive
N=10
22.68% Negative
N=213
76.25% Positive
N=716
Distribution of Ct values in cohort positive samples
64.39% Ct ≤ 25
11.59% 25 ≤ Ct ≤30
24.02% Ct > 30
Total=716
Prototype 1
Proof of concept
N=17
Negatives n=10
Positives N=7
Prototype 2
N=180
Negatives n=60
Positives N=120
Prototype 2
65.8% Ct ≤ 25
15.00% 25 ≤ Ct ≤30
19.20% Ct > 30
Prototype 3
N=180
Negatives n=60
Positives N=120
Prototype 3
70.00% Ct ≤ 25
12.50% 25 ≤ Ct ≤30
17.50% Ct > 30
[B]
Belo Horizonte
Follow-up
38 participants
112 positive samples
by RT-qPCR
0-3
Days
4-7
Days
>7
Days
N = 25(22.3%)
N = 45 (40.2%)
N = 42 (37.5%)
